# Supplementary material for: Irradiation by a Combination of Different Peak-Wavelength Ultraviolet-Light Emitting Diodes Enhances the Inactivation of Influenza A Viruses
Source: Microorganisms. 2020 Jul 8;8(7):1014. doi: 10.3390/microorganisms8071014 (PMC7409356; doi:10.3390/microorganisms8071014)
Supplement: Supplementary file 1 [file microorganisms-08-01014-s001.pdf]

microorganisms

Supplementary Materials

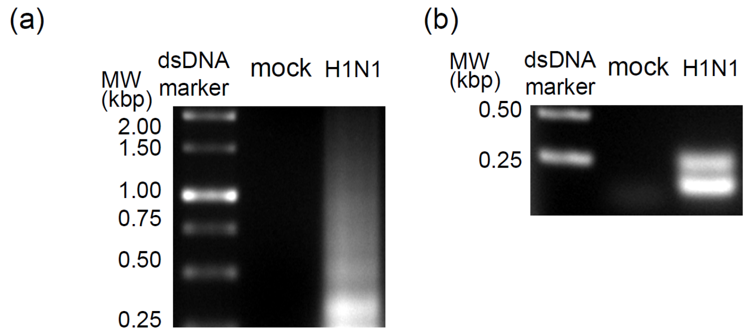

	mock	H1N1
RNA conc. (μg/mL)	6.7	103.5
A260/A280 ratio	1.03	1.93

Figure S1. Purification of viral RNA from allantoic fluids of mock- or H1N1 IAV-infected chicken embryonated eggs. The viral RNA was checked by agarose gel electrophoresis for 20 μL purified samples (a) and RT-PCR products for segment 4 (b), as described in Materials and Methods. Any pellets after ultracentrifusion and enough RNA concentration for gel electrophoresis were not observed in the mock-infected sample.
